# Anti-VEGF-Therapie bei fibrovaskulärer und serös-vaskularisierter Pigmentepithelabhebung bei neovaskulärer AMD: Eine retrospektive Fünf-Jahres-Analyse

**DOI:** 10.1007/s00347-020-01297-x

**Published:** 2020-12-15

**Authors:** T. Barth, M. Reiners, F. Zeman, R. Greslechner, H. Helbig, M.-A. Gamulescu

**Affiliations:** 1 Klinik und Poliklinik für Augenheilkunde, Universitätsklinikum Regensburg, Franz-Josef-Strauß-Allee 11, 93053 Regensburg, Deutschland; 2 Zentrum für klinische Studien, Universitätsklinikum Regensburg, Franz-Josef-Strauß-Allee 11, 93053 Regensburg, Deutschland

**Keywords:** Pigmentepithelabhebung, Optische Kohärenztomographie, Intravitreale Therapie, Langzeitverlauf, Real-life-Daten, Pigment epithelial detachment, Optical coherence tomography, Intravitreal therapy, Long-term outcome, Real-life data

## Abstract

**Hintergrund:**

Die neovaskuläre altersabhängige Makuladegeneration (nAMD) ist die häufigste Ursache für das Auftreten einer Pigmentepithelabhebung (PED). Die Therapie der fibrovaskulären PED (fPED) und serös-vaskularisierten PED (svPED) mit intravitrealen VEGF(„vascular endothelial growth factor“)-Inhibitoren hat in der klinischen Praxis eine eingeschränkte Prognose.

**Ziel der Arbeit:**

Die Datenlage zum PED-Langzeitverlauf ist überschaubar. Diese Arbeit erfasst den Verlauf therapierter PEDs bei nAMD über einen Zeitraum von 5 Jahren.

**Methodik:**

Bei allen Augen, die im Zeitraum von 2006 bis 2015 aufgrund einer fPED oder svPED eine Behandlung mit Anti-VEGF-Präparaten erhielten, erfolgte eine retrospektive Analyse des klinischen Verlaufs und der Morphologie mittels optischer Kohärenztomographie (OCT). Es galten die folgenden Einschlusskriterien: OCT-Nachweis einer PED, angiographische Bestätigung einer nAMD, klinische Dokumentation über 5 Jahre sowie gute Bildqualität.

**Ergebnisse:**

Bei 23 Augen von 22 Patienten wurden die Einschlusskriterien erfüllt. Nach 5 Jahren zeigte sich im Gesamtkollektiv und in der Subgruppe der Augen mit fPED eine signifikante Verschlechterung des Visus (*p* = 0,007; *p* = 0,045). Bei den Augen mit svPED war die Visusabnahme nicht signifikant (*p* = 0,097). Im Gesamtkollektiv wurde eine statistisch signifikante Reduktion der PED-Höhe (*p* = 0,006) bei gleichzeitig signifikanter Zunahme des PED-Durchmessers (*p* = 0,002) gemessen, wobei innerhalb der Subgruppen die Abnahme der PED-Höhe und Zunahme des PED-Durchmessers nur für die Messwerte der svPED signifikant waren (*p* = 0,004; *p* = 0,013). Bei den Augen mit fPED war die Veränderung der OCT-Parameter nicht signifikant (*p* = 0,616; *p* = 0,097). Bei 17 (74 %) der Augen fand sich bei der finalen OCT-Beurteilung eine Fibrose oder Atrophie.

**Diskussion:**

Nach 5 Jahren Anti-VEGF-Behandlung bei nAMD-assoziierter PED zeigten sich in der Hälfte der Fälle eine Visusabnahme und in drei Viertel der Augen eine ungünstige OCT-Morphologie. Die mittlere Anzahl an Injektionen und Visiten war niedriger als in klinischen Studien und anderen Real-life-Erhebungen. Insgesamt beobachteten wir eine Unterbehandlung mit schlechterem funktionellem und anatomischem Outcome in unserer klinischen Praxis verglichen mit anderen Studien.

## Abstract (en)

**Background:**

Neovascular age-related macular degeneration (nAMD) is the most frequent cause of pigment epithelial detachment (PED). In the clinical routine the treatment of fibrovascular PED (fPED) and serous vascularized PED (svPED) with intravitreal vascular endothelial growth factor (VEGF) inhibitors has a restricted prognosis.

**Objective:**

There are limited data on the long-term outcome of PED under anti-VEGF therapy. Therefore, this study recorded the course of treated PEDs in nAMD eyes over a period of 5 years.

**Methods:**

All eyes with fPED or svPED that underwent anti-VEGF medication between 2006 and 2015 were retrospectively analyzed regarding the clinical course and the morphology seen on optical coherence tomography (OCT). The inclusion criteria were the detection of a PED on OCT, the angiographic verification of nAMD, a documented clinical history over 5 years and a good image quality.

**Results:**

A total of 23 eyes from 22 patients met the inclusion criteria. After 5 years a significant deterioration of visual acuity (VA) was seen in all eyes (*p* = 0.007) and in the subgroup of cases with fPED (*p* = 0.045). In the eyes with svPED the decline of VA was not significant (*p* = 0.097). In the collective study group a statistically significant reduction of PED height (*p* = 0.006) and an increase of PED diameter was measured (*p* = 0.002). In the subgroup analysis the decrease of PED height and increase of PED diameter were significant for cases with svPED (*p* = 0.004, *p* = 0.013, respectively) but were not statistically significant for fPED eyes (height: *p* = 0.616; diameter: *p* = 0.097). In 17 (74%) eyes fibrosis or atrophy were seen on the final assessment of OCT images.

**Discussion:**

After 5 years of anti-VEGF therapy for nAMD-associated PED the VA declined in half of the eyes and the OCT showed an unfavorable morphology in 3/4 of the cases. The average number of visits and injections was distinctly lower than in clinical trials and other real-life analyses. In summary, we observed an undertreatment with a worse functional and anatomical outcome in our clinical routine compared to other studies.

Die Behandlung von Abhebungen des retinalen Pigmentepithels (PED) stellt im klinischen Alltag eine Herausforderung dar. Dabei ist die neovaskuläre altersabhängige Makuladegeneration (nAMD) die häufigste Ursache für das Auftreten einer PED. Der Anteil der Augen mit PED bei nAMD liegt bei ca. 10 % [3, 25]. Die Zulassungsstudien (ANCHOR, MARINA) zur „Anti-vascular endothelial growth factor“(VEGF)-Therapie bei nAMD liefern keine selektierten Daten zum klinischen Verlauf bei PED [2, 20]. Andere Autoren haben gezeigt, dass sich die PED im Langzeitverlauf und Ansprechen auf die Therapie von anderen Formen der nAMD unterscheidet [12, 24]. Diese Arbeit untersucht retrospektiv den Langzeitverlauf von Augen mit Anti-VEGF-Behandlung bei fibrovaskulärer und serös-vaskularisierter PED bei nAMD über 5 Jahre

## Hintergrund

Unter einer PED versteht man die Separation der Basalmembran des retinalen Pigmentepithels (RPE) von der darunter liegenden Bruch-Membran (BM) [22, 25]. Ätiologisch sind eine Dickenzunahme und Umschichtung der BM bedeutsam [25]. Durch die Ablagerung von Lipiden im Alter kommt es zur hydrophoben Diffusionsbarriere der BM mit schlechterer Passage von Stoffwechselprodukten zwischen Choroidea und RPE und zur Abnahme der Hydrokonduktivität. Durch die reduzierte Hydrokonduktivität der BM mit schlechterer Wasserpassage aus dem Glaskörperraum in Richtung Choroidea verbleibt Flüssigkeit unter dem RPE und führt zu einem „Hochpumpen“ des RPE [18, 25].

Morphologisch werden verschiedene Subtypen der AMD-assoziierten PED differenziert, wobei die Nomenklatur in der Literatur uneinheitlich ist. In der neuesten Konsensusempfehlung zur Nomenklatur der AMD werden bei den nichtneovaskulären AMD-Formen die drusenoide PED und die seröse PED (sPED) genannt. Gleichzeitig wird darauf hingewiesen, dass bei einer sPED häufig eine unerkannte neovaskuläre Komponente vorliegt, die durch multimodale Bildgebung abgeklärt werden sollte [22]. Die fibrovaskuläre PED (fPED) wird der nAMD zugeordnet [22]. Im deutschen Sprachraum ist die Unterscheidung in sPED, serös-vaskularisierte PED (svPED) und fPED gebräuchlich [6, 12]. Bei den beiden letztgenannten PED-Typen liegt in der Fluoreszeinangiographie (FA) oder Indozyaningrünangiographie (ICGA) eine choroidale Neovaskularisation (CNV) vor [26]. Nach neuer Nomenklatur spricht man auch von einer makulären Neovaskularisation (MNV) [22]. Während sich in der optischen Kohärenztomographie (OCT) bei der fPED eine flach ansteigende Elevation und eine hyperreflektive Binnenstruktur im Sub-RPE-Raum ohne seröse Anteile zeigen, findet sich bei den serösen PED-Formen eine steil ansteigende Elevation mit niedriger Binnenreflektivität [6, 26]. Eine rein seröse PED ohne nachweisbare CNV ist selten und sollte nur nach angiographischer Abklärung mittels FA und ICGA diagnostiziert werden [13, 22]. Die svPED zeigt ein Mischbild der Morphologie. Bei der retinalen angiomatösen Proliferation (RAP) und der polypoidalen choroidalen Vaskulopathie (PCV) besteht neben dem Vorliegen von PEDs eine charakteristische Morphologie und Klinik, sodass diese als eigene nAMD-Entitäten gelten und nicht in unsere Studie einbezogen wurden.

Die Behandlung der Wahl für die PED mit CNV-Nachweis ist die Therapie mit intravitrealen VEGF-Inhibitoren [12]. Eine typische Komplikation der PED ist hierbei der Einriss des RPE (PE-Riss), der ohne Therapie mit einer Wahrscheinlichkeit von ca. 10 % auftritt [3]. Diese Rate erhöht sich unter Anti-VEGF-Therapie auf ca. 15–27 % [5, 8].

## Ziel der Arbeit

Bisher ist die Datenlage zur intravitrealen Behandlung der PED bei nAMD überschaubar. In den Zulassungsstudien zur Anti-VEGF-Therapie erfolgte keine gesonderte Auswertung der Augen mit PED [2, 20]. In den wenigen Veröffentlichungen mit selektiver Analyse des klinischen Verlaufs von PED unter Therapie ist der Studienzeitraum mit 6 bis 24 Monaten recht kurz [23, 24]. Unsere Arbeit stellt die Auswertung des Langzeitverlaufs von Augen mit nAMD-assoziierter PED unter Anti-VEGF-Therapie vor.

## Methodik

Es wurden alle Fälle ermittelt, bei denen von 2006 bis 2015 (Therapiebeginn 2006 bis 2010) aufgrund einer fPED oder svPED eine Behandlung mit Anti-VEGF-Präparaten erfolgte. Die Einschlusskriterien waren der initiale Nachweis einer subfoveal gelegenen PED, definiert als Separation zwischen BM und RPE mit Bestätigung durch die Beurteilung zweier unabhängiger Retinologen, das Vorliegen einer angiographischen (FA und/oder ICGA) und OCT-Diagnostik bei Erstvorstellung mit Nachweis einer neovaskulären Komponente der Läsion, eine Therapie mit VEGF-Inhibitoren, eine Kontrolle des klinischen Verlaufs (Visus, OCT) über mindestens 60 Monate sowie eine ausreichend gute Bildqualität. Fälle mit einer drusenoiden PED ohne Neovaskularisation sowie Augen mit Typ-2-MNV, RAP oder PCV wurden von der Auswertung ausgeschlossen. Weiterhin wurden Fälle ausgeschossen, bei denen neben der Anti-VEGF-Therapie andere Behandlungsmodalitäten wie eine photodynamische Therapie (PDT) erfolgten. Ebenso wurden Patienten, bei denen anfangs ein Visus <0,05 vorlag oder morphologische Alterationen (Atrophie, Fibrose), bei denen keine Visusbesserung durch eine Therapie zu erwarten war, nicht evaluiert. Augen mit initialem PE-Riss wurden ebenfalls ausgeschlossen. Wurden die Einschlusskriterien erfüllt, erfolgte anhand der Krankenakten eine retrospektive Analyse der funktionellen und morphologischen Entwicklung der behandelten Augen über einen Beobachtungszeitraum von 5 Jahren. Erfasst wurden der Visusverlauf, die Anzahl der Visiten, die Häufigkeit der Injektionen und die Entwicklung der Läsion in der OCT. Zur Diagnostik gehörte bei allen Visiten eine ophthalmologische Untersuchung inklusive Ermittlung des bestkorrigierten Visus (BCVA), Spaltlampenbiomikroskopie und binokularer indirekter Ophthalmoskopie bei dilatierter Pupille. Für die statistische Auswertung wurden anhand der Akte die dezimalen Visuswerte vor Therapiebeginn, nach 6, 12 und nach 60 Monaten ermittelt und in LogMAR-Werte umgerechnet. Eine Fotodokumentation des Augenhintergrundes und eine Angiographie (Spectralis, Heidelberg Engineering) erfolgte immer bei der Erstvorstellung und bei Bedarf im Verlauf. Anhand der Angiographie wurde der Subtyp der CNV mit assoziierter PED klassifiziert (Typ 1 MNV nach Spaide) [22] und der größte lineare Durchmesser der neovaskulären Läsion (GLD) vermessen. Bei jeder Visite wurde eine Makula-OCT mit Darstellung der zentralen Netzhaut durch einen sternförmigen 6‑Zeilen-Scan durchgeführt (Spectral-domain/SD-OCT, Spectralis, Heidelberg Engineering). Die Analyse der Bilddaten erfolgte mit dem Heidelberg Eye Explorer (Heidelberg Engineering, Version 6.0.9.). Die Morphologie der Läsion (PED-Höhe, PED-Durchmesser) wurde anhand der OCT-Daten bei Erstdiagnose, nach 6, nach 12 und nach 60 Monaten evaluiert. Zwei unabhängige Retinologen führten die Klassifizierung des PED-Subtyps (svPED oder fPED) im Sinne der Konsensbildung sowie die Vermessung von PED-Höhe und PED-Durchmesser durch, wobei der Intraklassen-Korrelationskoeffizient (ICC) zur Beurteilung der Übereinstimmung berechnet wurde. Die PED-Höhe wurde als maximale vertikale Distanz zwischen BM und RPE-Basalmembran und der PED-Durchmesser als maximale horizontale Ausdehnung der Separation zwischen BM und RPE manuell ausgewertet. Zudem erfolgte anhand der Fundus- und OCT-Bilder eine deskriptive Beschreibung der finalen Netzhautsituation (Atrophie, Fibrose). Die intravitreale Therapie beinhaltete bei allen Augen anfangs die 3‑malige Gabe von Bevacizumab (Avastin®, Roche, 1,25 mg in 0,05 m), Ranibizumab 0,5 mg (Lucentis®; Novartis, 0,5 mg in 0,05 m) oder Aflibercept (Eylea®, Bayer, 2 mg in 0,05 m) in jeweils 4‑wöchigem Abstand. Nach diesem initialen Upload erfolgte in allen Fällen eine individualisierte Therapie je nach Bedarf („Pro-re-nata-Schema“ [PRN]). Weitere intravitreale operative Medikamenteneingaben (IVOM) erfolgten nach den allgemein anerkannten Behandlungskriterien der nAMD [11], wobei aufgrund langer Anfahrtswege unserer Patienten abweichend vom klassischen PRN-Schema eine Wiedereinbestellung im 4‑ bis 6‑wöchigen Intervall erfolgte.

Die statistische Auswertung erfolgte mit IBM SPSS® Statistics 25 (Armonk, NY, US). Quantitative Daten werden mit Mittelwert, Standardabweichung (SD) und Spannweite (Range) beschrieben, kategoriale Variablen als Anteile in Prozent. Der Vergleich der intervallskalierten Daten erfolgte bei verbundenen Stichproben mit dem t‑Test für gepaarte Stichproben und bei unverbundenen Stichproben mit dem Mann-Whitney-U-Test. Kategoriale Variablen wurden mithilfe des Chi-Quadrat-Tests für unabhängige Stichproben verglichen. In dieser explorativen Studie wurde ein *p*-Wert <0,05 für alle Analysen als statistisch signifikant gewertet.

## Ergebnisse

### Patientenselektion

Insgesamt wurden 183 Augen mit PED bei nAMD im Zeitraum von 2006 bis 2015 mit VEGF-Inhibitoren behandelt. Bei 99 (54 %) Krankheitsverläufen war der Beobachtungszeitraum von 5 Jahren unterbrochen oder nicht erfüllt. Bei 33 (18 %) von 183 Augen fand sich qualitativ unzureichendes oder veraltetes Bildmaterial im Time-Domain-Modus. Zwei (1 %) Fälle zeigten initial einen PE-Riss. Die Diagnose Typ-2-MNV (*n* = 2), RAP (*n* = 8) oder PCV (*n* = 10) führte bei 20 (11 %) Augen zum Ausschluss. Bei 2 (1 %) Augen lag rückblickend eine drusenoide PED ohne Neovaskularisation vor. In 4 (2 %) Fällen bestanden initial irreversible Netzhautveränderungen (Fibrose, alte Makulablutung), die zum Ausschluss führten. Lediglich in 23 (13 %) von 183 Fällen wurden die Einschlusskriterien erfüllt.

### Demografische Daten bei Erstdiagnose

Insgesamt erhielten 23 Augen von 22 Patienten, davon 14 Frauen (64 %) und 8 Männer (36 %), mit einer subfoveal gelegenen PED bei nAMD im Studienzeitraum eine Anti-VEGF-Therapie. Bei 1 männlichen Patienten mit svPED wurde im Verlauf eine beidseitige Therapie durchgeführt. Das mittlere Alter bei Therapiebeginn lag bei 74 Lebensjahren (SD 5,3; „range“ 64 bis 86 Jahre). Es wurden 12 (52 %) rechte Augen und 11 (48 %) linke Augen behandelt. Die mittlere Follow-up-Zeit lag bei 5,2 Jahren (SD 0,24; „range“ 4,98–5,85).

### Funktionelle Langzeitentwicklung

Vor Therapiebeginn betrug der mittlere LogMAR-Visus in der Studienpopulation 0,4 (SD 0,23; „range“ 0,2–1,1). Beim Vergleich der anfangs gemessenen Visusäquivalente mit den Werten nach 6 und 12 Monaten zeigte sich keine signifikante Veränderung des Visus (*p* = 0,812; *p* = 0,154), was als Stabilisierung der Visusentwicklung im 1. Therapiejahr gedeutet werden kann (Tab. 1). Nach insgesamt 5 Jahren zeigten 11 (49 %) Augen eine Visusverbesserung oder Stabilisierung des Visus, während bei 12 (52 %) Augen eine Visusverschlechterung erhoben wurde. Am Ende des Studienzeitraums zeigte sich ein durchschnittlicher LogMAR-Visus von 0,7 (SD 0,50; „range“ 0,2–2,2). Die Differenz zwischen initialem und finalem LogMAR-Visus nach 5 Jahren betrug im Mittel 0,3 (SD 0,50; „range“ −0,2 bis +1,8), was einem Visusverlust von ungefähr 3 Zeilen entspricht. Die Zunahme des LogMAR-Visus entsprechend einer funktionellen Verschlechterung war statistisch signifikant (*p* = 0,007). Die Tab. 1 gibt einen Überblick über die wichtigsten klinischen Daten der Studienpopulation (Tab. 1).

**Table 1:** 

Klinische Langzeitdaten von 23 Augen von 22 Patienten mit PED	
Anzahl der Augen	23
Rechtes Auge	12 (52 %)
Weibliches Geschlecht (%)	14 (61 %)
Alter bei Erstdiagnose (Jahre)	74 (SD 5,3)
Beobachtungszeitraum (Jahre)	5,2 (SD 0,24)
Anteil Typ-1-MNV nach Angiographie	23 (100 %)
GLD (µm) der MNV	2842 (SD 873,8)
Anzahl der Visiten	17,7 (SD 8,27)
Anzahl der Anti-VEGF-Injektionen	16,0 (SD 11,52)
LogMAR BCVA initial	0,4 (SD 0,23)
LogMAR BCVA nach 6 Monaten	0,4 (SD 0,23)
LogMAR BCVA nach 1 Jahr	0,5 (SD 0,33)
LogMAR BCVA nach 5 Jahren	0,7 (SD 0,50)
PED-Höhe initial (µm)	376 (SD 261,3)
PED-Höhe nach 6 Monaten (µm)	370 (SD 316,0)
PED-Höhe nach 1 Jahr (µm)	358 (SD 233,4)
PED-Höhe nach 5 Jahren (µm)	255 (SD 164,4)
PED-Durchmesser initial (µm)	2867 (SD 1161,5)
PED-Durchmesser nach 6 Monaten (µm)	2927 (SD 1187,8)
PED-Durchmesser nach 1 Jahr (µm)	3314 (SD 1399,7)
PED-Durchmesser nach 5 Jahren (µm)	3941 (SD 1489,6)

*PED* Pigmentepithelabhebung, *BCVA* „best corrected visual acuity“/bester korrigierter Visus, *SD* Standardabweichung, *VEGF* „vascular endothelial growth factor“, *MNV* makuläre Neovaskularisation, *GLD* größter linearer Durchmesser

### Morphologische Langzeitentwicklung

Angiographisch fand sich bei allen Augen eine Typ-1-MNV. Der durchschnittliche GLD lag in der FLA bei 2842 (SD 873,8; „range“ 1195–4331). Anhand der OCT zeigte sich bei Erstvorstellung in 10 (43 %) Fällen eine fPED und in 13 (57 %) Augen eine svPED. Zwischen initialer PED-Morphologie (Höhe, Durchmesser) und Ausgangsvisus bestand kein signifikanter Zusammenhang (r = 0,04 und *p* = 0,854; r = 0,24 und *p* = 0,264). Innerhalb von 5 Jahren nahm die PED-Höhe unter Anti-VEGF-Therapie von anfangs 376 µm (SD 261,6; „range“ 120–1074) auf 255 µm (SD 164,4; „range“ 67–764) ab. Diese Reduktion der mittleren PED-Höhe war statistisch signifikant (*p* = 0,006). Der maximale horizontale Durchmesser der PED zeigte eine signifikante Zunahme von anfangs 2867 µm (SD 1161,5; „range“ 633–5865) auf 3941 µm (SD 1489,6; „range“ 1504–6631) bei der finalen Auswertung (*p* = 0,002). Bei der deskriptiven finalen Netzhautbefundung zeigte sich bei 15 (65 %) Augen ein als „Narbe“ oder „Fibrose“ beurteilter OCT-Befund. Bei 2 (9 %) Augen wurde der Endbefund als geografische Atrophie (GA) beschrieben. Fünf (22 %) Augen zeigten persistierende sub- oder intraretinale Flüssigkeit ohne erkennbare Fibrose, während bei 1 Auge ein trocknerer Netzhautbefund vorlag.

### Anzahl der Visiten

Insgesamt wurden im Verlauf von 5 Jahren pro Auge im Mittel 17,7 Visiten (SD 8,27; „range“ 5–30) mit Visusprüfung und OCT-Kontrolle dokumentiert. Dies entspricht durchschnittlich 3,5 Visiten pro Jahr und Auge. Während die mittlere Visitenanzahl im 1. Jahr bei 5,3 (SD 1,29; „range“ 3–8) lag, sank dieser Wert in den Folgejahren auf durchschnittlich 2,3 bis 3,5 Visiten pro Jahr (Tab. 2). Diese Abnahme zwischen Visitenzahl im 1. Jahr war verglichen mit dem 2. bis 5. Jahr jeweils statistisch signifikant (*p* = 0,03; *p* < 0,001; *p* < 0,001; *p* < 0,001).

**Table 2:** 

Monitoring und Anti-VEGF-Therapie bei 23 Augen von 22 Patienten mit PED						
	1. Jahr	2. Jahr	3. Jahr	4. Jahr	5. Jahr	Gesamt
Anzahl der Visiten	5,3 (SD 1,29)	3,4 (SD 2,61)	2,3 (SD 2,32)	2,9 (SD 2,33)	3,5 (SD 1,90)	17,7 (SD 8,27)
Anzahl der Injektionen	4,8 (SD 1,76)	2,8 (SD 2,86)	2,5 (SD 2,98)	2,5 (SD 2,78)	3,3 (SD 3,36)	16,0 (SD 11,52)

*PED* Pigmentepithelabhebung, *VEGF* „vascular endothelial growth factor“

### Anzahl der Injektionen

Pro Auge wurden innerhalb von 5 Jahren durchschnittlich 16,0 (SD 11,52; „range“ 3–40) Injektionen mit einem VEGF-Inhibitor durchgeführt. Zwischen initialer PED-Morphologie (Höhe, Durchmesser) und Anzahl der Injektionen bestand kein signifikanter Zusammenhang (r = 0,00 und *p* = 0,996; r = 0,24 und *p* = 0,272). Im 1. Behandlungsjahr erfolgten mit durchschnittlich 4,8 (SD 1,76; „range“ 1–8) IVOM die meisten intravitrealen Anti-VEGF-Eingaben. Danach lag die mittlere Anzahl der Injektionen bei 2,5 bis 3,3 IVOM pro Jahr (Tab. 2). Die Reduktion der Injektionszahl im 2. bis 4. Jahr war verglichen mit dem 1. Jahr statistisch jeweils signifikant (*p* < 0,001; *p* < 0,001; *p* < 0,001). Zwischen dem 1. und 5. Jahr bestand kein Unterschied in der mittleren Anzahl der Injektionen (*p* = 0,057).

Bei den Augen mit Visusverschlechterung am Ende des Beobachtungszeitraums (*n* = 12, 52 %) fand sich im Vergleich zu der Gruppe mit gebessertem oder stabilem Visus (*n* = 11, 48 %) kein signifikanter Unterschied in der Anzahl der Kontrollvisiten (stabil/besser: 17 Visiten, SD 8,7; „range“ 5–27 vs. schlechter: 19 Visiten, SD 8,0; „range“ 8–30) (*p* = 0,497). Ebenso wenig zeigte sich ein Zusammenhang zwischen der Injektionsanzahl und der Zugehörigkeit zur Gruppe Visusverschlechterung vs. Visusstabilisierung/-besserung (*p* = 0,689). Betrachtet man gesondert die Augen mit einer intensiven Anti-VEGF-Therapie (≥25 Injektionen in 5 Jahren, *n* = 5), findet sich kein signifikanter Unterschied in der Visusdifferenz im Vergleich zu den anderen Fällen (*p* = 0,308). Bezüglich des Therapiemonitorings und der intravitrealen Anti-VEGF-Behandlung zeigte sich zwischen den Augen mit fPED und svPED kein Unterschied (Tab. 3). Die Anzahl der Visiten war in der Subgruppe der fPED und svPED vergleichbar (*p* = 0,576). Dasselbe gilt für die Injektionsanzahl, bei der sich zwischen beiden PED-Subtypen kein statistisch signifikanter Unterschied fand (*p* = 0,535). Die Augen mit fPED erhielten im Mittel 15,5 IVOM (SD 14,05; „range“ 3–40), während in den Fällen mit svPED 16,3 Anti-VEGF-Gaben (SD 9,74; „range“ 3–36) erfolgten.

**Table 3:** 

PED-Subtyp	fPED	svPED
Anzahl der Augen	10 (43 %)	13 (57 %)
Rechtes Auge	5 (50 %)	7 (54 %)
Weibliches Geschlecht (%)	7 (70 %)	7 (54 %)
Alter bei ED (Jahre)	74 (SD 6,0)	74 (SD 4,9)
LogMAR BCVA initial	0,4 (SD 0,13)	0,5 (SD 0,28)
LogMAR BCVA nach 6 Monaten	0,4 (SD 0,15)	0,5 (SD 0,28)
LogMAR BCVA nach 12 Monaten	0,5 (SD 0,31)	0,5 (SD 0,36)
LogMAR BCVA nach 5 Jahren	0,8 (SD 0,59)	0,7 (SD 0,44)
Anzahl der Visiten	17 (SD 7,7)	18 (SD 9,0)
Anzahl der Anti-VEGF-Injektionen	16 (SD 14,1)	16 (SD 9,7)

*PED* Pigmentepithelabhebung, *fPED* fibrovaskuläre PED, *svPED* serös-vaskularisierte PED, *ED* Erstdiagnose, *BCVA* „best corrected visual acuity“/bester korrigierter Visus, *SD* Standardabweichung, *VEGF* „vascular endothelial growth factor“

### PE-Risse

Bei 4 (17 %) Augen entwickelte sich im Verlauf ein PE-Einriss. Bei 3 (75 %) der Fälle trat der Einriss innerhalb des ersten Halbjahres auf, beim 4. Patienten erst im letzten Beobachtungsjahr. In 3 (75 %) der Fälle mit PE-Riss lag eine svPED vor. Die mittlere Anzahl der Injektionen (13,5; SD 9,47, „range“ 3–22) unterschied sich nicht signifikant zwischen Augen mit und ohne PE-Riss (*p* = 0,597). Ebenso wenig unterschieden sich der mittlere finale Visus (0,8; SD 0,48) und die mittlere Visusdifferenz (0,23; SD 0,37) dieser Fälle von den Augen ohne PE-Riss (*p* = 0,410; *p* = 0,837). Es bestand kein Zusammenhang zwischen initialer PED-Höhe und dem Auftreten eines PE-Risses, wobei aufgrund der kleinen Kohorte die detaillierte Analyse der Subgruppe mit PE-Riss nicht aussagekräftig ist.

### Anti-VEGF-Präparate

Die Mehrzahl der Augen erhielt eine Monotherapie mit Ranibizumab (*n* = 18, 78 %). Bei den Fällen mit Therapiebeginn im Jahr 2006 erfolgte anfangs eine intravitreale Off-label-Behandlung mit Bevacizumab, die im Jahr 2007 nach der Zulassung von Lucentis® für die nAMD auf Ranibizumab umgestellt wurde (*n* = 4, 17 %). Bei 1 (4 %) Auge erfolgte im Verlauf bei persistierender Flüssigkeit eine Therapieumstellung von Ranibizumab auf Aflibercept.

### PED-Subgruppenanalyse

Insgesamt wurden 10 (43 %) Augen mit fPED und 13 (57 %) Augen mit svPED ausgewertet (Tab. 3). Die Alters- und Geschlechterverteilung wies in beiden Gruppen keinen signifikanten Unterschied auf (Alter: *p* = 0,732; Geschlecht: *p* = 0,442). Bezüglich des Visus zeigten sich bei den Augen mit fPED und svPED ähnliche initiale Visuswerte (*p* = 0,242). Im weiteren Beobachtungszeitraum ließen sich keine Unterschiede der Visusentwicklung zwischen beiden Gruppen feststellen (nach 6 Monaten: *p* = 0,660, nach 12 Monaten: *p* = 0,594 nach 5 Jahren: *p* = 1,0).

### Funktionelle Entwicklung der PED-Subtypen

In der Subgruppe der fPED blieb der initiale LogMAR BCVA von im Mittel 0,4 (SD 0,13; „range“ 0,2–0,6) im 1. Jahr stabil (*p* = 0,078) und zeigte nach 5 Jahren eine Zunahme auf durchschnittlich 0,8 (SD 0,59; „range“ 0,2–2,2), was einer funktionellen Verschlechterung von ungefähr 4 Visuszeilen entspricht. In der Gruppe der svPED zeigten sich im 1. Jahr ein stabiler Visus sowie nach 5 Jahren eine Zunahme des LogMAR BCVA von anfangs 0,5 (SD 0,28; „range“ 0,2–1,1) auf 0,7 (SD 0,44; „range“ 0,2–1,5) entsprechend einer mittleren Visusverschlechterung von ungefähr 2 Zeilen. Der Unterschied zwischen initialem und finalem Visus war in der Gruppe der fPED signifikant (*p* = 0,045) und bei den Augen mit svPED nicht signifikant (*p* = 0,097).

### Morphologische Entwicklung der PED-Subtypen

In Tab. 4 werden die mittels OCT gemessenen morphologischen Langzeitdaten der Augen mit fPED und svPED vergleichend aufgelistet (Tab. 4). Bei der OCT-basierten manuellen Vermessung der beiden PED-Subtypen durch 2 unabhängige Untersucher betrug der ICC für die Höhe 0,998 (95 %-CI: 0,997; 0,999) und für den Durchmesser 0,987 (95 %-CI: 0,980; 0,992). Weder vor Therapiebeginn noch nach 5 Jahren Anti-VEGF-Therapie bestand ein signifikanter Unterschied zwischen der PED-Höhe beider Gruppen (initial: *p* = 0,321; final: *p* = 0,710). Innerhalb der Gruppe der svPED zeigte sich eine statistisch signifikante Reduktion der PED-Höhe von anfangs 440 µm (SD 300,0; „range“ 109–800) auf final 240 µm (SD 146,3; „range“ 58–538) (*p* = 0,004), bei gleichzeitig stabilem Verlauf der PED-Höhe in der Gruppe der fPED (*p* = 0,616). Der PED-Durchmesser nahm in der Gruppe der svPED innerhalb des Beobachtungszeitraums von anfangs 2635 µm (SD 1422,1; „range“ 633–5865) auf 3844 µm (SD 1427,2; „range“ 1814–5614) signifikant zu (*p* = 0,013). Bei den Augen mit fPED war die Zunahme des Durchmessers der Läsion von initial 3170 µm (SD 649,1; 2386–4146) auf final 4067 µm (SD 1636,1; „range“ 1504–6243) statistisch nicht signifikant (*p* = 0,097). Zwischen den beiden Gruppen zeigte sich hinsichtlich des PED-Durchmessers zu keinem Zeitpunkt der Auswertung ein signifikanter Unterschied (initial: *p* = 0,121; nach 6 Monaten: *p* = 0,264; nach 12 Monaten: *p* = 0,756, nach 5 Jahren: *p* = 0,756).

**Table 4:** 

PED-Subtyp	fPED	svPED
Anzahl der Augen	10 (43 %)	13 (57 %)
PED-Höhe initial (µm)	293 (SD 183,7)	440 (SD 300,0)
PED-Höhe nach 6 Monaten (µm)	357 (SD 377,7)	380 (SD 275,3)
PED-Höhe nach 1 Jahr (µm)	280 (SD 189,0)	417 (SD 253,5)
PED-Höhe nach 5 Jahren (µm)	274 (SD 191,9)	240 (SD 146,3)
PED-Durchmesser initial (µm)	3170 (SD 649,1)	2635 (SD 1422,1)
PED-Durchmesser nach 6 Monaten (µm)	3254 (SD 1179,4)	2676 (SD 1177,5)
PED-Durchmesser nach 1 Jahr (µm)	3345 (SD 1250,0)	3291 (SD 1554,5)
PED-Durchmesser nach 5 Jahren (µm)	4067 (SD 1636,1)	3844 (SD 1427,2)

*PED* Pigmentepithelabhebung, *fPED* fibrovaskuläre PED, *svPED* serös vaskularisierte PED, *SD* Standardabweichung

**Figure 1:**
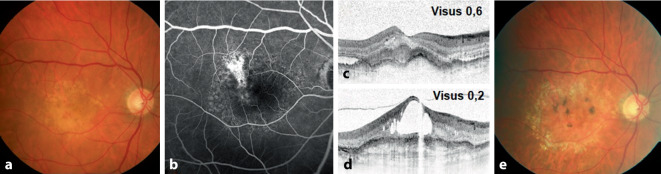

**Figure 2:**
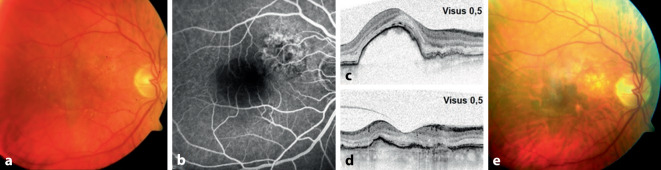

Insgesamt fand sich bei 7 Augen mit fPED (70 %) und bei 8 Augen mit svPED (77 %) am Ende des Studienzeitraums ein fibrotischer Netzhautbefund. In Abb. 1 und 2 sind exemplarisch die klinischen Verläufe von einem Fall mit fPED (Abb. 1) und svPED (Abb. 2) dargestellt.

## Diskussion

Es wurden Langzeitdaten von insgesamt 23 Augen von 22 Patienten mit einer intravitrealen Anti-VEGF-Behandlung bei fPED und svPED bei nAMD retrospektiv ausgewertet.

Insgesamt zeigte sich nach 5 Jahren intravitrealer Anti-VEGF-Therapie eine statistisch signifikante Reduktion des Visus in der Gesamtpopulation (*p* = 0,007). In der Subgruppe der fPED war diese funktionelle Verschlechterung signifikant (*p* = 0,045), während die Visusabnahme bei den Augen mit svPED nicht signifikant war (*p* = 0,097).

### Langzeitdaten zur Anti-VEGF-Therapie bei nAMD

Zuletzt veröffentlichten einige Autoren die Langzeitergebnisse zur Anti-VEGF-Therapie bei nAMD mit 5 bis 7 Jahren Beobachtungszeit [1, 14, 15, 17, 19]. Ein Vergleich dieser Ergebnisse mit unseren Daten ist nicht möglich, da bei diesen Studien Risikoformen wie die PED ausgeklammert, ihr Anteil nicht genau beziffert wurde oder nur PEDs mit einem 50 %igen CNV-Anteil eingeschlossen wurden [2, 19, 20]. Wichtig ist, zwischen Daten aus klinischen Studien und Real-life-Erhebungen zu unterscheiden. Maguire et al. zeigten in den 5‑Jahres-Daten der CATT-Studie (Comparison of Age-related Macular Degeneration Treatments Trials), dass unter Real-life-Bedingungen die innerhalb von klinischen Studien erzielten Visusgewinne durch die Anti-VEGF-Therapie der nAMD nach Beendigung des Protokolls trotz Fortführung der Behandlung nicht gehalten werden konnten [17]. Zu ähnlichen Resultaten kam die SEVEN-UP-Studie, bei der die im Rahmen der Zulassungsstudien (ANCHOR und MARINA) erzielten Visusbesserungen nach Beendigung des Protokolls in den 7‑Jahres-Daten nicht gehalten werden konnten [19]. Betrachten wir den morphologischen Verlauf, zeigen Langzeitauswertungen in Übereinstimmung mit unseren Daten (in 65 % Fibrose) die langfristig ungünstige anatomische Entwicklung von Augen mit nAMD unter Anti-VEGF-Therapie. Die SEVEN-UP-Studie zeigte nach 7 Jahren Anti-VEGF-Therapie bei 61 % der Augen eine makuläre Fibrose und bei 39 % eine subfoveale Fibrose, während Gillies et al. bei 31 % der behandelten Augen nach 7 Jahren eine Fibrose fanden [14, 19]. Die Anzahl an Injektionen (6,8 IVOM über eine Periode von 3,4 Jahren) reflektiert eine deutliche Untertherapie in der Real-life-Situation [19]. Airody et al. zeigten in einer Gruppe von britischen Patienten (*n* = 68) anhand von 5‑Jahres-Daten zur Ranibizumab-Behandlung bei nAMD unter einem PRN-Regime bei einer mittleren Injektionsanzahl von 4 bis 7 Injektionen pro Jahr bessere Visusergebnisse mit einer Stabilisierung ohne Visusverlust nach 5 Jahren [1]. Der Zusammenhang zwischen Anzahl der Injektionen und stabilem Visusverlauf wurde auch in anderen Erhebungen festgestellt. In einer belgischen Kohorte (*n* = 69) wurde mittels PRN-Schemas bei einer mittleren Injektionsanzahl von 2,5 bis 5,2 Ranibizumab-Gaben pro Jahr kein signifikanter Visusverlust nach 6 Jahren festgestellt [15]. In unserer Kohorte lag die mittlere Anzahl bei 2,5 bis 4,8 IVOM pro Jahr, was mit den Daten von Jacob vergleichbar ist, aber unter den britischen Zahlen liegt [1]. Bei Gillies et al. fanden sich in einer australischen 7‑Jahres-Auswertung (*n* = 131) ebenfalls höhere Raten mit einer mittleren Anzahl von 6,1 Injektionen im 1. Jahr und 4,9 bis 5,5 IVOM im 2. bis 7. Jahr, unter denen eine Visusstabilisierung erreicht wurde [14]. Betrachtet man das Monitoring, so war in der britischen Analyse (9 bis 10 Kontrollen pro Jahr), in der australischen Studie (7,4 bis 8,7 Kontrollen pro Jahr) und in der belgischen Auswertung (6,9 bis 10,3 Kontrollen pro Jahr) die Anzahl der Visiten deutlich höher als in unserer Erhebung (2,3 bis 5,3 Kontrollen pro Jahr) [1, 14, 15]. Insgesamt war die mittlere Anzahl an Injektionen und Visiten in unserem Kollektiv deutlich niedriger als in klinischen Studien und geringer als in anderen Real-life-Erhebungen, sodass diskutiert werden muss, ob unsere Ergebnisse durch ein intensiveres Kontroll- und Therapieregime positiver ausgefallen wären. Sachs et al. wiesen in ihrer Arbeit auf die Bedeutung der Adhärenz der Patienten als Voraussetzung für den langfristigen Therapieerfolg und eine Visusstabilisierung hin [21]. Wie bereits bei unserer Patientenselektion aufgefallen, war bei 99 von 183 (54 %) Krankheitsverläufen der Studienzeitraum von 5 Jahren lückenhaft oder nicht abgeschlossen. Daher sollten Patienten bereits bei der Erstdiagnose auf die Bedeutung konsequenter Kontrollen als Voraussetzung für den langfristigen Visuserhalt hingewiesen werden. Zudem sollte auch der behandelnde Arzt die Verantwortung für ein konsequentes Therapiemonitoring übernehmen. Zusammenfassend sahen wir in unserer klinischen Praxis verglichen mit Studienbedingungen eine Unterbehandlung mit ungünstigem funktionellem und anatomischem Outcome.

### Langzeitdaten zur Anti-VEGF-Therapie bei neovaskulärer PED

Die speziell zur Anti-VEGF-Therapie bei neovaskulärer PED verfügbaren Studien sind überschaubar und mit relativem kurzem Follow-up (6 Monate bis 2 Jahre) [4, 16, 23, 24]. Tran et al. untersuchten in einer retrospektiven, multizentrischen Studie den Verlauf von 44 Augen mit Aflibercept-Therapie bei PED bei nAMD über einen Zeitraum von 2 Jahren [23]. Hierbei zeigte sich nur in den ersten 3 bis 6 Monaten eine signifikante Visusbesserung bei gleichzeitiger PED-Höhen-Reduktion, während bei der finalen 2‑Jahres-Auswerung kein signifikanter Unterschied von Visus und PED-Höhe feststellbar war [23]. In der Arbeit von Vaze et al. zeigten sich bei 92 Patienten mit PED nach 6 Monaten ein signifikanter Visusanstieg sowie eine Reduktion der PED-Höhe, wobei kein Unterschied zwischen einer Therapie mit Ranibizumab oder Aflibercept festgestellt wurde [24]. Die prospektive, multizentrische RECOVER-Studie zeigte nach 1 Jahr monatlicher Ranibizumab-Therapie einen signifikanten Visusanstieg bei 29 Augen mit svPED und eine funktionelle Stabilisierung bei 11 Augen mit fPED [9]. In unserer Erhebung zeigte sich ebenfalls nach 6 und 12 Monaten eine Visusbesserung, jedoch nach 5 Jahren und damit nach einem deutlich längeren Beobachtungszeitraum als in der RECOVER-Studie eine signifikante funktionelle Verschlechterung (Zunahme des LogMAR-Visus) im Gesamtkollektiv (*p* = 0,007) und in der Subgruppe der fPED (*p* = 0,045). Augen mit svPED zeigten keine signifikante Visusreduktion (*p* = 0,097). Dieses bessere Abschneiden der Augen mit svPED im funktionellen Langzeitverlauf könnte, wie es auch Clemens et al. in der Diskussion der RECOVER-Daten vermuten, dem differenten morphologischen Verlauf zugeschrieben werden [9]. Während nämlich übereinstimmend mit den RECOVER-Daten die Augen mit svPED unter Anti-VEGF-Therapie eine signifikante Reduktion der PED-Höhe zeigten (*p* = 0,004), wurde bei den Fällen mit fPED keine signifikante Höhenverringerung der PED gemessen (*p* = 0,616). Dies entspricht auch den Ergebnissen von Clemens et al. bei 18 Augen mit vaskularisierter PED nach 1 Jahr monatlicher Aflibercept-Therapie, bei denen eine signifikante PED-Höhen-Abnahme bei jedoch unverändertem Visus gemessen wurde [7]. Zur PED-Höhe ist anzumerken, dass bei der svPED aufgrund der Morphologie (steilere Elevation mit durchschnittlich höherer initialer PED-Höhe und größerem serösem Anteil) ein größeres Potenzial für eine Reduktion der PED-Höhe besteht. Weiterhin stellt laut Clemens et al. der große CNV-Anteil bei der fPED ein mechanisches Hindernis für eine PED-Abflachung dar [6]. Die Häufigkeit des Auftretens von PE-Rissen entspricht den Ergebnissen anderer Autoren [3, 8]. In 75 % kam es innerhalb der ersten 6 Monate zum Einriss des RPE, wobei vermutet wird, dass die intravitreale Anti-VEGF-Gabe eine Kontraktion der neovaskulären Membran begünstigt, die sekundär zum PE-Riss führt [5, 8, 10]. Allen zitierten Arbeiten gemeinsam ist der im Gegensatz zu unserer Analyse relativ kurze Follow-up-Zeitraum von maximal 2 Jahren, sodass Aussagen zur langfristigen funktionellen und morphologischen Entwicklung nicht abzuleiten sind.

### Limitationen

Trotz des recht langen Beobachtungszeitraums unserer Studie von 5 Jahren ist limitierend anzumerken, dass es sich um eine retrospektive, monozentrische Auswertung einer kleinen Kohorte ohne Kontrollgruppe handelt. Zudem zeigte sich in unserem Kollektiv eine deutlich niedrigere Visiten- und Injektionsrate als in anderen Studien. Dies erklären wir uns durch den vom klassischen PRN-Schema abweichenden Wiedereinbestellmodus von 4 bis 6 Wochen sowie durch individuelle Faktoren (Krankheit, Terminverschiebungen, fehlende Adhärenz) und strukturelle Parameter (fehlendes Recall-System). Kritisch anzumerken ist, dass die Werte der final gemessenen OCT-Parameter nicht exakt, sondern nur annähernd bestimmt werden konnten, da in der OCT-Bildgebung nach 5 Jahren ausgeprägte morphologische Alterationen die exakte Vermessung der PED erschwerten. Weiterhin lässt sich aufgrund der kleinen Fallzahl und der heterogenen Gruppengröße keine Aussage zur Wirksamkeit der verschiedenen Anti-VEGF-Präparate treffen. Positiv ist, dass sich unsere Arbeit auf die fPED und svPED als spezielle Formen der nAMD bezieht, zu deren klinischem Verlauf unter Anti-VEGF-Therapie es bisher nur wenige Real-life-Erhebungen mit langem Follow-up-Zeitraum gibt.

## Fazit für die Praxis

- Nach 5 Jahren Anti-VEGF-Therapie wies die Hälfte (52 %) der Augen mit nAMD-assoziierter PED eine Visusverschlechterung auf.
- Drei Viertel der Augen (74 %) zeigten morphologisch ungünstige Befunde wie eine Fibrose oder GA.
- In der Subgruppe der Augen mit svPED wurde nach 5 Jahren keine signifikante Visusverschlechterung beobachtet. Bei den Fällen mit fvPED zeigte sich ein signifikanter Visusabfall.
- Die PED-Höhe nahm bei Augen mit svPED signifikant ab, während bei den Fällen mit fPED keine signifikante Reduktion der Höhe gemessen wurde.
- Die mittlere Anzahl an Visiten und Injektionen war deutlich geringer als in klinischen Studien und niedriger als in anderen Real-life-Analysen.
- In der klinischen Praxis kann eine Unterbehandlung im Vergleich zu kontrollierten Studienbedingungen vorliegen.
- Im Alltag der nAMD-Therapie sollten Arzt und Patient gleichermaßen ihren Beitrag zu einem konsequenten Therapiemonitoring leisten.
